# P-1307. High-dose versus Standard-dose Ampicillin-Sulbactam for Acinetobacter baumannii Infections

**DOI:** 10.1093/ofid/ofaf695.1495

**Published:** 2026-01-11

**Authors:** Emily Hou, Evan Steere, Eric Gregory

**Affiliations:** The University of Kansas Health System, Visalia, California; The University of Kansas Health System, Visalia, California; The University of Kansas Health System, Visalia, California

## Abstract

**Background:**

The 2024 Infectious Diseases Society of America Guidance on the Treatment of Antimicrobial-Resistant Gram-Negative Infections recommends high-dose ampicillin-sulbactam (SAM) as part of combination therapy for carbapenem-resistant Acinetobacter baumannii-calcoaceticus complex (ABC) infections when sulbactam-durlobactam is unavailable. The purpose of this study was to evaluate the safety and efficacy of high-dose SAM (> 9 grams/day of sulbactam) vs. standard-dose SAM (< 9 grams/day of sulbactam) in treating various ABC infections.

**Figure:**
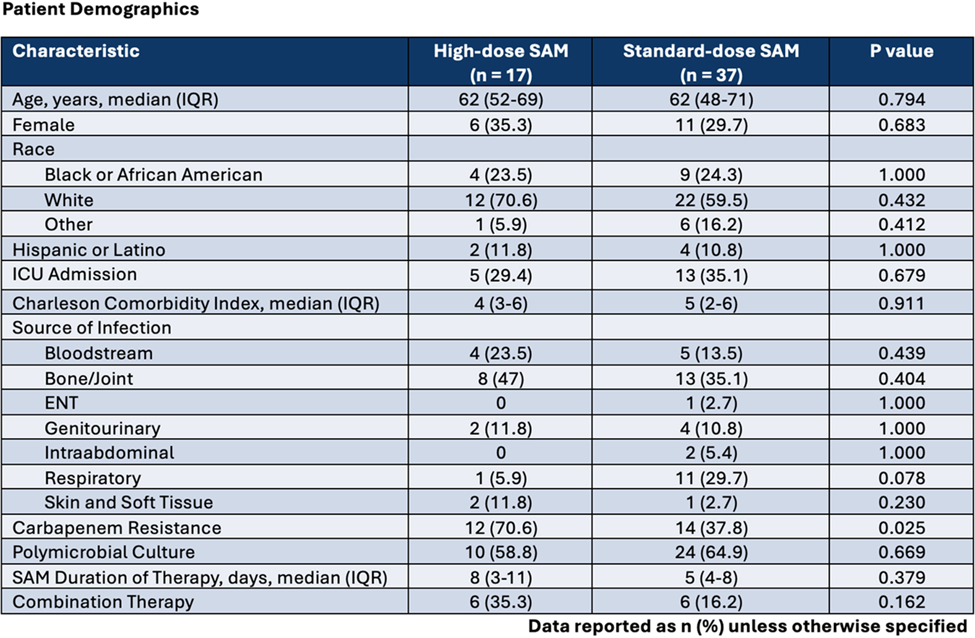

**Figure:**
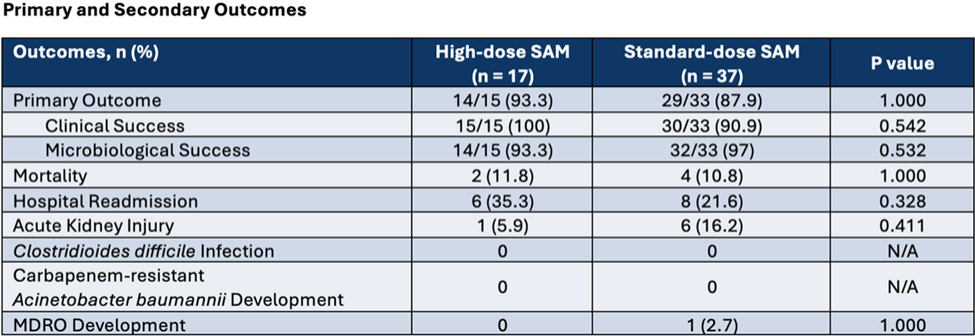

**Methods:**

This was an IRB-approved, single-center, retrospective cohort study of adult patients admitted to The University of Kansas Health System with an index positive ABC isolate susceptible to SAM that was treated with > 3 days of SAM between 10/2020 and 10/2024. Exclusion criteria included pregnancy, death or discharge to hospice prior to receiving > 3 days of SAM, or repeat ABC isolates within study period. The primary endpoint was meeting both clinical success (improvement of signs/symptoms of index infection per treating physician) and microbiological success (no recurrence of initial/alternative infection with ABC within 30 days after SAM was stopped). Secondary endpoints included 30-day all-cause mortality, hospital readmission, occurrence of acute kidney injury (AKI), Clostridioides difficile infection, and development of antimicrobial resistance.

**Results:**

A total of 240 patients were screened, of which 54 were included in the study. More patients received standard (68.5%) compared to high-dose SAM (31.5%). The most common sources of infection were bone/joint (38.9%), respiratory (22.2%), and bloodstream (16.7%). Carbapenem-resistance was phenotypically present in 26 (48.1%) ABC isolates. Most infections in the standard-dose group were polymicrobial. However, concurrent ABC-targeted combination therapy was the same in both groups. There was no statistical difference in the primary outcome of microbiological and treatment success between the high vs. standard-dose SAM groups (93.3% vs. 87.9%, p = 1.000). No difference in 30-day all-cause mortality, AKI, or development of resistance were identified.

**Conclusion:**

Our study showed no difference in treatment success with standard vs high-dose SAM in various ABC infections.

**Disclosures:**

All Authors: No reported disclosures

